# Polygenic risk for autism spectrum disorder affects left amygdala activity and negative emotion in schizophrenia

**DOI:** 10.1038/s41398-020-01001-2

**Published:** 2020-09-21

**Authors:** Yue Qin, Jujiao Kang, Zeyu Jiao, Yi Wang, Jiucun Wang, Hongyan Wang, Jianfeng Feng, Li Jin, Fei Wang, Xiaohong Gong

**Affiliations:** 1 State Key Laboratory of Genetic Engineering, School of Life Sciences, Fudan University, Shanghai, China; 2 Shanghai Center for Mathematical Science, Fudan University, Shanghai, China; 3 Human Phoneme Institute, Fudan University, Shanghai, China; 4 Obstetrics and Gynecology Hospital, Fudan University, Shanghai, China; 5 Institute of Science and Technology for Brain-inspired Intelligence, Fudan University, Shanghai, China; 6 Department of Computer Science, University of Warwick, Coventry, CV4 7AL UK; 7 Department of Radiology, The First Affiliated Hospital of China Medical University, Shenyang, China

**Keywords:** Molecular neuroscience, Medical genetics, Psychiatric disorders

## Abstract

Although the diagnoses based on phenomenology have many practical advantages, accumulating evidence shows that schizophrenia and autism spectrum disorder (ASD) share some overlap in genetics and clinical presentation. It remains largely unknown how ASD-associated polygenetic risk contributes to the pathogenesis of schizophrenia. In the present study, we calculated high-resolution ASD polygenic risk scores (ASD PRSs) and selected optimal ten ASD PRS with minimal *P* values in the association analysis of PRSs, with schizophrenia to assess the effect of ASD PRS on brain neural activity in schizophrenia cases and controls. We found that amplitude of low-frequency fluctuation in left amygdala was positively associated with ASD PRSs in our cohort. Correlation analysis of ASD PRSs with facial emotion recognition test identified the negative correlation of ASD PRSs with negative emotions in schizophrenia cases and controls. Finally, functional enrichment analysis of PRS genes revealed that neural system function and development, as well as signal transduction, were mainly enriched in PRS genes. Our results provide empirical evidence that polygenic risk for ASD contributes to schizophrenia by the intermediate phenotypes of left amygdala function and emotion recognition. It provides a promising strategy to understand the relationship between phenotypes and genotypes shared in mental disorders.

## Introduction

Schizophrenia and autism have historically been considered as related diagnostic categories. Earlier, autism was viewed as the childhood onset of schizophrenia^1^ or a central feature of schizophrenia^2^. The term autism was used interchangeably with schizophrenia until the 1970s, when Kolvin and Rutter highlighted their differences^3,4^. Autism spectrum disorder (ASD) and schizophrenia co-occur at a higher rate than expected in the general population. In populations with schizophrenia spectrum disorders, rates of comorbid ASD were reported from 3.6 to 60%, much higher than the prevalence of 1% for ASD in the general population, and vice versa, as reviewed by Chisholm et al.^5^.

Accumulating evidence shows that schizophrenia and ASD share some overlap in genetic risk factors^6–9^. A large number of particular copy number variations (CNVs) and monogenic abnormalities are implicated in both disorders, including 22q11.2, 1q21.1, 15q13.3, *DISC1*, *RELN*, *SHANK3*, *BDNF*, *FOXP2*, *NLGN3*, *NRCAM*, *CACNA1C*, and *GRIN2B*^10^. Their function is associated with neurodevelopment and synaptic plasticity, which sheds light on common mechanisms between two disorders. A milestone study performed by Psychiatric Genomics Consortium showed a significant genetic correlation between schizophrenia and ASD^11^, implying a modest overlap of common genetic variation. Impaired social cognition is a core feature of clinical presentations of schizophrenia and ASD^12–15^. Individuals with schizophrenia as well as those with ASD evidence impaired emotional function, especially deficits in facial affect recognition, which is a necessary prerequisite for the understanding of other’s intentions^16^. Impairments in face recognition are consistently associated with ASD from early period of life to adult^17–21^. Recent research carried out with children across Israel, Britain, and Sweden suggests that recognition deficits in emotions appear cross-cultural, indicating a universal nature in the ASD population^22^. A meta-analysis showed that emotion perception impairment represents a robust finding in schizophrenia^23^, and this aberration has been examined to remain across cultures^24^.

There is increasing awareness that the genetic architecture of schizophrenia and ASD is polygenic. Findings from genome-wide association studies (GWASs) indicate that multiple common genetic variants of small effect contribute to the etiology of schizophrenia^25–30^ and ASD^6,31–33^. In recent years, polygenic risk scores (PRSs) have been widely used in investigating the polygenic architecture of complex disorders^34–38^. PRS measures the additive effects of hundreds or thousands of nominally associated common variants identified through large-scale GWAS and may capture polygenic architecture of a given disorder or trait. The application of PRS strategy in imaging genetics provides opportunities to investigate the neuroimaging markers influenced by genetic risk, and thus improves understanding of cognitive and emotional processes, and the neural mechanism of mental disorders^39–42^.

Resting-state functional magnetic resonance imaging (rs-fMRI), a technique that measures spontaneous low-frequency fluctuations in the blood oxygenation level-dependent signal (BOLD) in the brain, is uniquely suitable for mental disorder patients because it is noninvasive and does not require a task to perform. The amplitude of low-frequency fluctuation (ALFF) is an efficient index that detects the regional intensity of spontaneous LFFs (0.01–0.08 Hz)^43^ in BOLD signal and is thought to reflect spontaneous neural function of the brain^44^. In 2007, ALFF algorithm was first used in clinical research^43^. Although the exact neural substrate for ALFF is unclear, ALFF shows high reliability and sensitivity in gray matter regions^45–48^, and may act as a biomarker of differences between groups and individuals^43,48^. Many ALFF studies in schizophrenia have shown significant differences compared with health controls. Overall, increased ALFF was found primarily in the right putamen, right inferior frontal gyrus, left inferior temporal gyrus, and right anterior cingulate cortex, while decreased ALFF was noted mainly in the bilateral postcentral gyrus, bilateral precuneus, left inferior parietal gyri, and right occipital lobe in schizophrenia^49^. In addition, in a recent ALFF study in three major psychiatric disorders (schizophrenia, bipolar disorder, and major depressive disorder), common alterations in ALFF patterns were observed across three disorders in the neural system for emotional perception, such as increased ALFF in the striatal, limbic, paralimbic, and heteromodal regions, and decreased ALFF in the visual cortex^50^.

As mentioned above, schizophrenia and ASD share multiple common genetic variants and clinical characteristics, but it remains largely unknown how ASD-associated genetic variants ultimately contribute to the development of schizophrenia. To explore this mystery, we calculated ASD PRS, and tested its relationship with ALFF and facial emotion recognition test in schizophrenia cases and controls. The functional enrichment analysis was performed to annotate the biological significance of PRS genes.

## Materials and methods

### Participants

The total sample of 345 participants consisted of patients with schizophrenia (*n* = 121) and healthy controls (HC; *n* = 224). Patients with schizophrenia were recruited from the inpatient and outpatient services at Shenyang Mental Health Center and Department of Psychiatry, First Affiliated Hospital of China Medical University, Shenyang, China. HC participants were recruited from local community by advertisement. All patients were diagnosed by two trained psychiatrists independently using the Structured Clinical Interview for DSM-IV Axis I Disorders in participants 18 years or older and the Schedule for Affective Disorders and Schizophrenia for School-Age Children-Present and Lifetime Version (K-SADS-PL) in those younger than 18. All Patients met Diagnostic and Statistical Manual of Mental Disorders, Fourth Edition (DSM-IV) diagnostic criteria for schizophrenia, and no other comorbid Axis I disorder. Exclusion was applied to the patients if any of the following were present: (1) the presence of a concomitant major medical disorder, (2) history of seizures, head trauma, or unconsciousness for greater than or equal to 5 min, (3) history of substance/alcohol abuse or dependence, and (4) any contraindications to MRI. HC participants did not have current or lifetime DSM-IV Axis I disorder or history of psychotic, mood, or other DSM-IV Axis I disorders in first-degree relatives, as determined by a detailed family history. This study was approved by the Institutional Review Board of China Medical University. All participants were required to sign an informed consent after receiving a detailed description of the study.

Clinical symptoms of all participants with schizophrenia were evaluated by the 17-item Hamilton Depression Rating Scale, the Hamilton Anxiety Scale, and the Brief Psychiatric Rating Scale.

### Facial emotion recognition test

A facial emotion recognition test was conducted with the Chinese Facial Affective Picture System^51^, consisting of gray-scale images for six basic types of facial expressions (anger, disgust, fear, sadness, surprise, and happiness) and neutral ones. Participants were asked to identify emotional expressions from these photographs. Each face photograph was displayed on the computer screen for 6–7 s, followed by an interval of 1–2 s. Three conditions derived from these emotion pictures were recorded: positive, negative, and neural. If the judgement was true, one point was scored for the corresponding group. After finishing the test, total points of the three groups for each participant were summarized independently as three values. The sum of three groups was also calculated as total value.

### Genotyping and imputation

Genomic DNA was extracted from whole blood using standard protocols. The samples were genotyped on the Illumina Global Screening Array-24 v1.0 BeadChip. This array provides data for 642,824 fixed genetic variants, in addition to 53,411 customized variants. Single-nucleotide polymorphisms (SNPs) with minor allele frequency < 1%, call rate < 95% or Hardy–Weinberg equilibrium *P* < 10^−5^ were excluded from the analysis. Individuals with excessive missingness > 5%, gender mismatch, or an estimation of identity-by-descent > 0.90 were also excluded from the study. Genotype imputation was performed by a commercial imputation engine named GenoImpute^52^. A mean sample-level *r*^2^ of 0.736 estimated by 1% hold out SNPs on the array was obtained. Different with other off-the-shelf imputation engines, this engine produces a continuous allele dosage, as well as three genotype probability distribution that reflects the reality of genotype uncertainty. The allele dosage was used for later analysis.

### Calculation of PRSs

We used the latest international GWAS results published in Nature Genetics^53^, consisting of 18,381 ASD cases and 27,969 controls, as discovery sample and our imputed genotyping data, as target sample. PRS was generated using PRSice software (www.PRSice.info)^54^. We performed *P* value-informed clumping with a cutoff of *r*^2^ = 0.1 in a 250-kb window. In order to avoid the deviation caused by manual selection of ASD PRS at a few *P* value thresholds (*P*_T_), 103 *P*_T_ (ranging from 0 to 0.5 with increments of 0.005, plus 10^−5^, 10^−4^, and 10^−3^) were computed for each individual. Population stratification was assessed and the first four components generated by PLINK were used as covariates in the later analyses.

Subsequently, ASD PRS was divided into two subsets: ASD&SCZ PRS with SNPs shared with schizophrenia, and ASD-specific PRS with SNPs only detected in ASD at certain *P*_T_ values. Polygenic risk for schizophrenia (SCZ PRS) was defined from the discovery sample, comprised of 33,426 SCZ cases and 32,541 controls, in a GWAS paper published in Cell^55^.

### MRI data acquisition and processing

All participants underwent functional and anatomical data acquisition on a clinical 3-T MRI scanner (GE Signal HD) with a standard eight-channel head coil at the First Affiliated Hospital of China Medical University, Shenyang, China. Individuals were instructed to keep eyes closed and remain awake during our study. Functional MR data were acquired using a single-shot echo-planar imaging sequence (repetition time 2000 ms, echo time 40 ms, field of view (FOV) 240 × 240 mm^2^, in-plane matrix 64 × 64, and flip angle 90°). Thirty-five axial slices were collected with 3 mm thickness without gap. Also, high-resolution 3D T1-weighted anatomical images were acquired using a 3-D fast spoiled gradient-echo sequence (TR/TE = 7.1/3.2 ms, image matrix = 240 × 240, FOV = 240 × 240 mm^2^, 176 contiguous slices of 1 mm without gap, voxel size = 1.0 mm^3^).

Functional image processing was conducted as described previously^56,57^. Briefly, the functional images were processed with SPM8 (http://www.fil.ion.ucl.ac.uk/spm) and DPARSF (http://www.restfmri.net/forum/DPARSF)^58^. For each participant, the first ten volumes of scanned data were discarded due to instability of the initial signal. Each participant’s motion was assessed by means of translation/rotation, and an exclusion criterion (translation >3 mm, rotation >3° in each direction) was set. The realigned functional data were then normalized to the standard EPI template in Montreal Neurological Institute space, and resampled to 3 × 3 × 3 mm^3^. Images were spatially smoothed with a 6 mm full-width at half-maximum Gaussian kernel. Linear regression and temporal band-pass filtering (0.01–0.08 Hz) were used to reduce the effects of low-frequency drifts and physiological high-frequency physiological noise. Nuisance signals, including six head motion parameters, global mean signal, white matter signal and cerebrospinal fluid signal, were regressed out from the data. ALFF at each voxel was calculated as the averaged square root of the power in the above frequency windows normalized by the mean within-brain ALFF value for each subject. The automated anatomical labeling (AAL) atlas, which partitioned the brain into 90 regions of interest (45 in each hemisphere) was used to obtain the region-wise ALFF.

### Statistical analyses

When appropriate, *t* test and chi-square test were used to test for differences between individuals, with schizophrenia and HC for demographic and clinical variables.

Association of PRSs with case–control status was performed with logistic regression in PRSice, and Nagelkerke’s pseudo-*R*^2^ was calculated to measure the proportion of variance. To correct for multiple tests, a significance threshold of *P* = 0.001 was adopted as suggested by Euesden et al.^54^.

Statistical analyses were conducted to assess the association of certain variables with PRS using multiple linear regression modeling in Matlab software. Variables examined in ALFF analysis was region-wise ALFF of 90 brain regions of AAL atlas, and the covariates included in this model were age, sex, education, disease status, and the first four components for population stratification. Variables of interest in clinical phenotypes analysis were four values (positive, negative, neutral, and total) of facial expressions, and this model included age, sex, education, disease status, and the first four components for population stratification as covariates. Correction for multiple comparisons was made using Bonferroni with *P*_corrected_ < 0.05. To examine whether the relationship between the variable of interest and PRS differs between cases and controls, we tested the interaction between PRS and disease status on the variable of interest in the regression model with age, sex, education, and the first four components for population stratification as covariates.

### Gene functional enrichment analyses

All the SNPs under certain *P*_T_ of PRS were extracted from the discovery sample. Then the gene list corresponding to these SNPs was identified according to the database of dbSNP. Gene Ontology (GO) terms and Kyoto Encyclopedia of Genes and Genomes (KEGG) pathway analyses of genes of PRS were performed with the Database for Annotation, Visualization, and Integrated Discovery (DAVID) Bioinformatics Resources version 6.8 (https://david.ncifcrf.gov/)^59,60^. Annotations were limited to *Homo sapiens*. The function of a particular gene was annotated with three GO terms: biological process (BP), cellular component (CC), and molecular function (MF). Bonferroni procedure was performed for multiple testing with significance threshold <0.01.

## Results

### Sample characteristics

Five schizophrenia cases and 12 HC were excluded during the quality control procedures of genotyping and imputation. Finally, 116 schizophrenia cases and 212 HC were included in the present study. The controls were older and had higher education level compared with schizophrenia patients (Table 1). During the following statistical analysis, age, sex, and education were considered as covariates.

**Table 1:** Participant characteristics.

	Schizophrenia	Healthy control	*t*/*χ*^2^	*P*
	(*n* = 116)	(*n* = 212)		
Demographic characteristics				
Age at scan(year, mean ± SD)	24.24 ± 9.03	34.71 ± 12.69	−7.8606	<0.001
Gender (M/F)	36/80	83/129	2.1365	0.1438
Handedness(R/L/MIX)	103/2/ 11	197/3/11	2.2433	0.3257
Education(year, mean ± SD)	10.78 ± 2.92	13.76 ± 3.71	−7.4394	<0.001
Clinical characteristics				
First episode(yes/no)	66/49	NA		
Medication(yes/no)	96/19	NA		
Duration (months, mean ± SD)	29.96 ± 48.91	NA		
HAMD-17	*n* = 113	*n* = 211		
	6.44 ± 6.28	1.15 ± 1.94	11.2953	<0.001
HAMA	*n* = 112	*n* = 212		
	6.13 ± 6.27	1.13 ± 2.00	10.6606	<0.001
BPRS	*n* = 115	*n* = 197		
	31.56 ± 11.80	18.28 ± 1.66	15.5367	<0.001
CFAPS	*n* = 83	*n* = 184		
Positive (*n*, mean ± SD)	11.25 ± 0.0841	11.55 ± 0.0256	0.9052	0.3670
Neutral (*n*, mean ± SD)	13.45 ± 0.1846	14.74 ± 0.0299	2.805	0.006
Negative (*n*, mean ± SD)	11.98 ± 0.1366	13.20 ± 0.0327	2.977	0.0035
Total (*n*, mean ± SD)	36.67 ± 0.6459	39.51 ± 0.1083	3.259	0.0015

*P* refers to the *P* value, calculated from chi-square test (gender and handedness) or *t* test (other variables) between two groups.

*SD* standard deviation, *M* male, *F* female, *R* right, *L* left, *MIX* left and right, *HAMD-17* the 17-item Hamilton Depression Rating Scale, *HAMA* the Hamilton Anxiety Scale, *BPRS* the Brief Psychiatric Rating Scale, *CFAPS* the Chinese Facial Affective Picture System.

### Selection of optimal ASD PRS that contributed to schizophrenia mostly

**Fig. 1:**
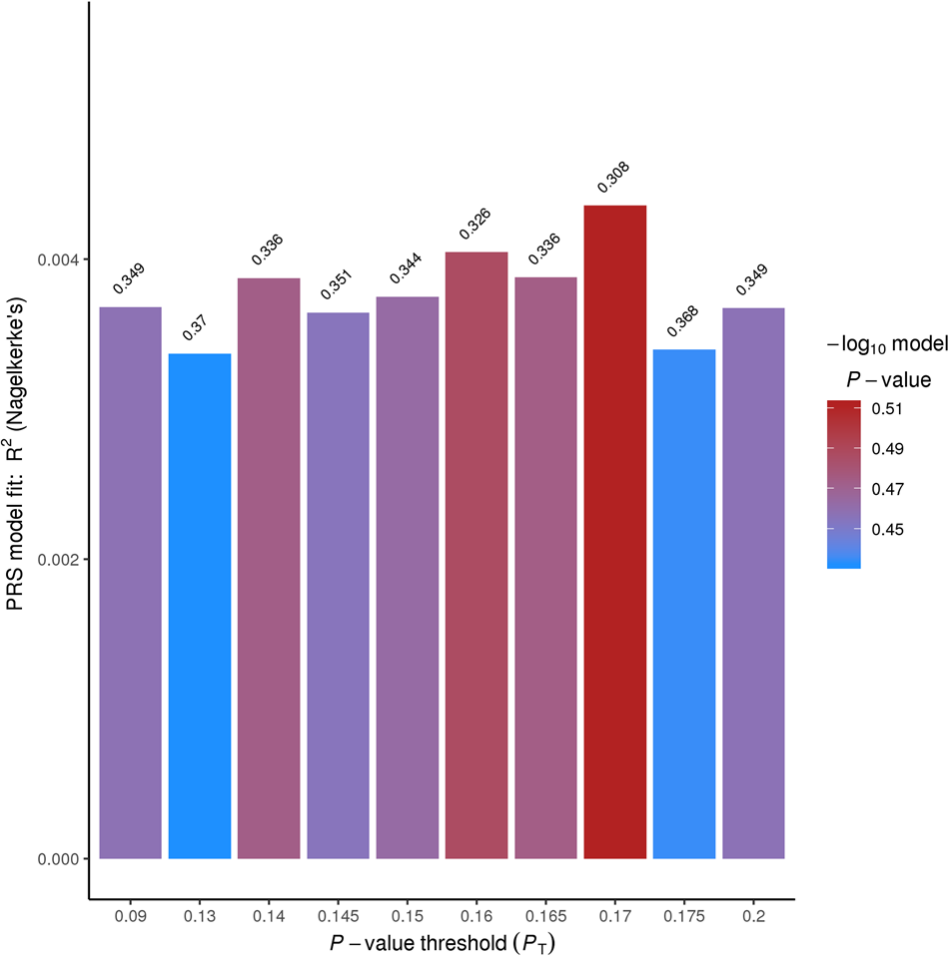
Top ten ASD PRS in the association analysis of PRS with schizophrenia. The *x*-axis represents *P* value threshold (*P*_T_), and *y*-axis represents Nagelkerke’s pseudo-*R*^2^, which means the variance of case–control status explained by PRS.

A total of 103 ASD PRS at high-resolution *P*_T_ were analyzed in schizophrenia cases and controls to select optimal ASD PRS scores, which contributed to schizophrenia mostly. Optimal ten ASD PRS scores with minimal *P* values at *P*_T_ of 0.09, 0.13, 0.14, 0.145, 0.15, 0.16, 0.165, 0.17, 0.175, and 0.2 were obtained (Fig. 1), though there was no significant difference between schizophrenia cases and controls for ASD PRS.

### Effect of ASD PRS on ALFF

**Fig. 2:**
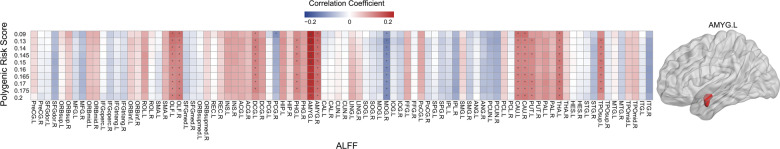
Associations between ASD PRS and ALFF values of 90 AAL brain regions. Brain regions which showed the correlation with ASD PRS were marked with asterisks. *P_uncorrected_ < 0.05, ×*P*_uncorrected_ < 0.00055 (significant after Bonferroni correction). The most significant results were obtained in the left amygdala. The color bar indicates correlation coefficient from negative (blue) to positive (red). Location of left amygdala in the human brain was shown on the right panel.

Multiple linear regression analysis revealed a positive correlation between ASD PRS and ALFF in left amygdala in the total sample of schizophrenia cases and controls (Fig. 2). The strongest association was observed at ASD PRS at *P*_T__0.13 (*r* = 0.2122, *P*_uncorrected_ = 0.0002483, *P*_corrected_ = 0.022). All ten tested PRS showed a correlation with ALFF in left amygdala (*P*_uncorrected_ < 0.05) and five of those remained significant after Bonferroni correction. We did not find significant interaction between ASD PRS and illness status on ALFF in left amygdala. These results indicated that the effect of ASD PRS on ALFF in left amygdala was robust and not influenced by disease status.

There were also tendencies of correlation of ASD PRS scores with ALFF in other regions like left and right olfactory cortex and the paralimbic cortices, including left median cingulate and paracingulate gyri, and left temporal pole of superior temporal gyrus (*P*_uncorrected_ < 0.05; Supplementary Table S1).

### Correlations of ASD PRS with facial emotion recognition

**Fig. 3:**
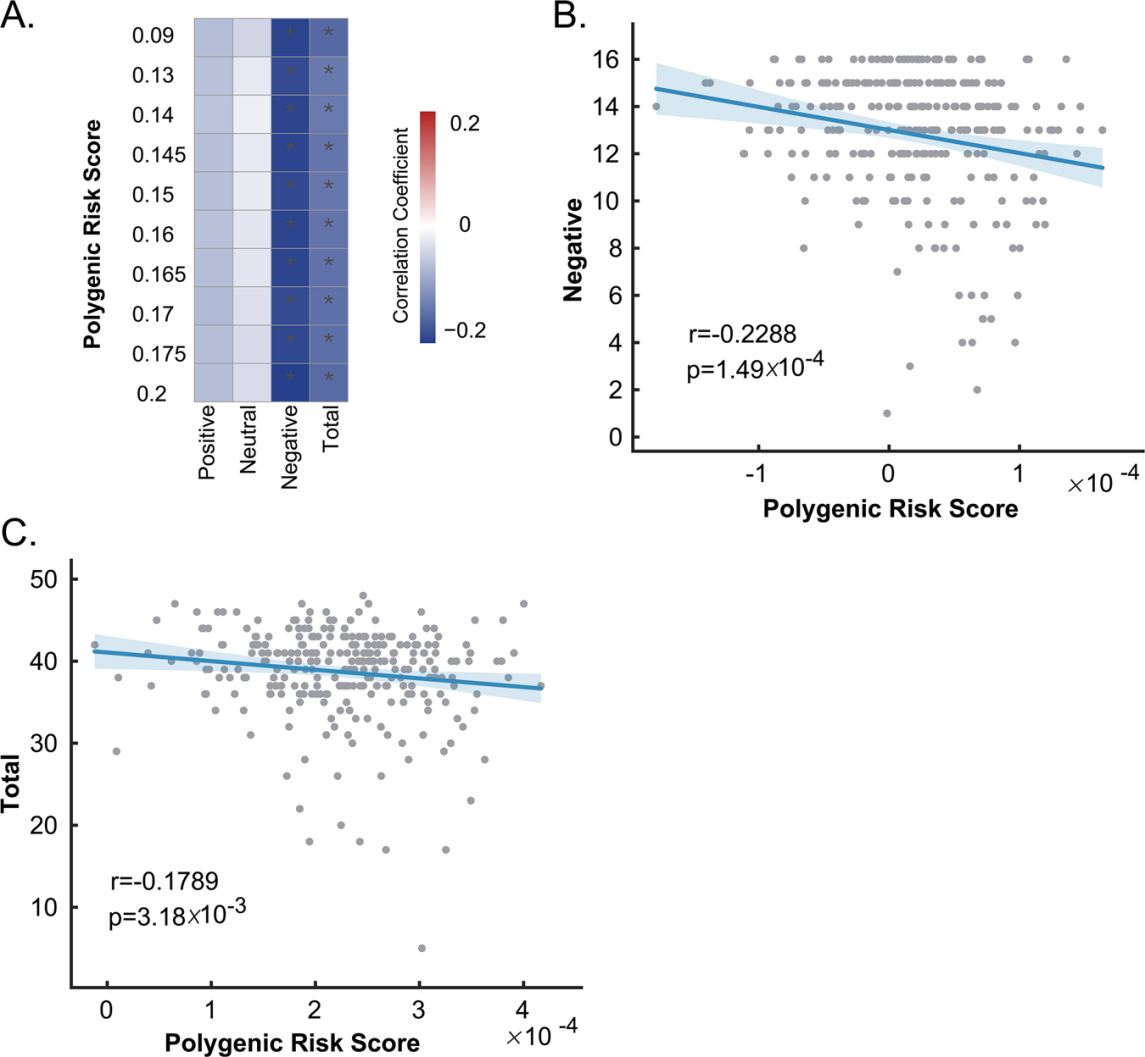
Correlations of ASD PRS with facial emotion recognition. **a** Multiple linear regression assessment showed that ASD PRS was negatively correlated with negative emotions and total value. The color bar indicates correlation coefficient from negative (blue) to positive (red). *P_uncorrected_ < 0.05. **b** The scatterplot showed the negative correlation of ASD PRS with negative emotions at *P*_T__0.2 (*r* = −0.2288, *P*_uncorrected_ = 0.000149). **c** The scatterplot showed the negative correlation of ASD PRS with total value at *P*_T__0.09 (*r* = −0.1789, *P*_uncorrected_ = 0.00318).

Considering the important role of amygdala in emotion recognition, we assessed the correlation of ASD PRS with facial emotion recognition test in schizophrenia cases and controls (Fig. 3a). Negative correlation of ASD PRS with negative emotions were found, of which the strongest association was obtained at *P*_T__0.2 (*r* = −0.2288, *P*_uncorrected_ = 0.000149, *P*_corrected_ = 0.0006; Fig. 3b). ASD PRS at other nine *P*_T_ values also showed significant association with the negative emotions after multiple testing corrections, providing evidence that the influence of ASD PRS on perception of negative emotions was consistent and robust. ASD PRS also showed negative correlation with total value at ten *P*_T_ values and remained significance after Bonferroni correction. The strongest association was obtained at *P*_T__0.09 (*r* = −0.1789, *P*_uncorrected_ = 0.00318, *P*_corrected_ = 0.013; Fig. 3c). Disease status was adjusted when we carried out the analysis of the correlation between ASD PRS and facial emotion recognition test. To further explore if disease status influences this correlation, we compared the slopes of the regression lines of ASD PRS and measures in facial emotion recognition test between schizophrenia cases and controls, and did not find significant difference. So, there was no significant interaction between ASD PRS and illness status on facial emotion recognition results.

### ASD-specific PRS affects ALFF in left amygdala and negative emotion recognition

In ASD PRS, there are two components considering the SNPs under certain *P*_T_ values: SNPs shared with schizophrenia (corresponding to ASD&SCZ PRS) and SNPs specific to ASD (corresponding to ASD-specific PRS). ASD PRS has been proven correlated with ALFF in left amygdala and negative emotions. In order to test if schizophrenia related common variants mediates this correlation, we investigated the relationships between three sets of PRSs (SCZ PRS, ASD&SCZ PRS, and ASD-specific PRS), and ALFF in left amygdala and negative emotions at same ten *P*_T_ values (Table 2). Intriguingly, ASD-specific PRS was positively correlated with ALFF in left amygdala and negatively correlated with recognition in negative emotions, consistent with the results of ASD PRS while showing lower *P* values. The strongest association between ASD-specific PRS and ALFF in left amygdala was obtained at *P*_T__0.09 (*r* = 0.2224, *P*_uncorrected_ = 0.000120, *P*_corrected_ = 0.0108;). The strongest association between ASD-specific PRS and negative emotions recognition was obtained at *P*_T__0.09 (*r* = −0.2749, *P*_uncorrected_ = 0.00000454, *P*_corrected_ = 0.0000182). Neither SCZ PRS nor ASD&SCZ PRS was associated with these measures. These findings suggested that ASD PRS, especially ASD-specific PRS, influenced ALFF in left amygdala and negative emotions recognition.

**Table 2:** Results from multiple linear regression analyses between four sets of PRS and ALFF in left amygdala, or performance in facial emotion recognition test.

PRS at *P*_T_	ALFF in left amygdala				Positive emotions				Neutral emotions				Negative emotions				Total			
	ASD^a^	SCZ	ASD&SCZ	ASD specific	ASD^b^	SCZ	ASD&SCZ	ASD specific	ASD^b^	SCZ	ASD&SCZ	ASD specific	ASD^b^	SCZ	ASD&SCZ	ASD specific	ASD^b^	SCZ	ASD&SCZ	ASD specific
0.09	2.66E−04	0.860	0.155	1.20E−04	0.211	0.737	0.811	0.233	0.416	0.161	0.614	0.260	2.12E−04	0.848	0.576	4.54E−06	3.18E−03	0.302	0.502	4.12E−04
0.13	2.48E−04	0.855	0.431	4.10E−04	0.230	0.825	0.508	0.195	0.680	0.186	0.542	0.387	3.91E−04	0.776	0.844	1.15E−05	8.69E−03	0.495	0.473	8.03E−04
0.14	4.55E−04	0.773	0.285	1.00E−03	0.255	0.703	0.838	0.287	0.758	0.178	0.244	0.511	2.44E−04	0.726	0.344	1.17E−05	9.17E−03	0.462	0.222	1.74E−03
0.145	3.65E−04	0.809	0.227	7.52E−04	0.225	0.628	0.850	0.251	0.683	0.167	0.200	0.439	2.27E−04	0.790	0.305	1.36E−05	6.83E−03	0.398	0.180	1.32E−03
0.15	4.20E−04	0.780	0.124	4.25E−04	0.213	0.671	0.949	0.242	0.697	0.166	0.321	0.408	3.21E−04	0.807	0.403	2.59E−05	7.69E−03	0.403	0.307	1.46E−03
0.16	7.45E−04	0.842	0.111	4.53E−04	0.226	0.681	0.969	0.181	0.631	0.218	0.414	0.405	2.42E−04	0.686	0.434	1.59E−05	6.31E−03	0.504	0.381	8.88E−04
0.165	5.97E−04	0.789	0.186	5.81E−04	0.207	0.670	0.927	0.154	0.624	0.297	0.457	0.354	2.52E−04	0.639	0.207	1.59E−05	5.85E−03	0.592	0.271	6.38E−04
0.17	6.13E−04	0.828	0.137	9.32E−04	0.181	0.651	0.787	0.122	0.560	0.317	0.363	0.341	3.75E−04	0.633	0.145	3.61E−05	5.27E−03	0.600	0.171	6.85E−04
0.175	8.11E−04	0.788	0.128	2.67E−03	0.213	0.624	0.494	0.163	0.495	0.264	0.341	0.279	3.05E−04	0.707	0.074	2.61E−05	4.63E−03	0.510	0.081	5.87E−04
0.2	1.42E−03	0.806	0.067	4.36E−03	0.209	0.752	0.248	0.226	0.508	0.196	0.153	0.191	1.49E−04	0.719	0.026	1.14E−05	3.57E−03	0.488	0.015	3.77E−04

*ASD* ASD PRS, *SCZ* SCZ PRS, *ASD&SCZ* ASD&SCZ PRS, *ASD specific* ASD-specific PRS.

^a^The *P* values for ASD PRS are exactly the values in Fig. 2.

^b^The *P* values for ASD PRS are exactly the values in Fig. 3a.

### Functional annotation of ASD PRS genes

**Fig. 4:**
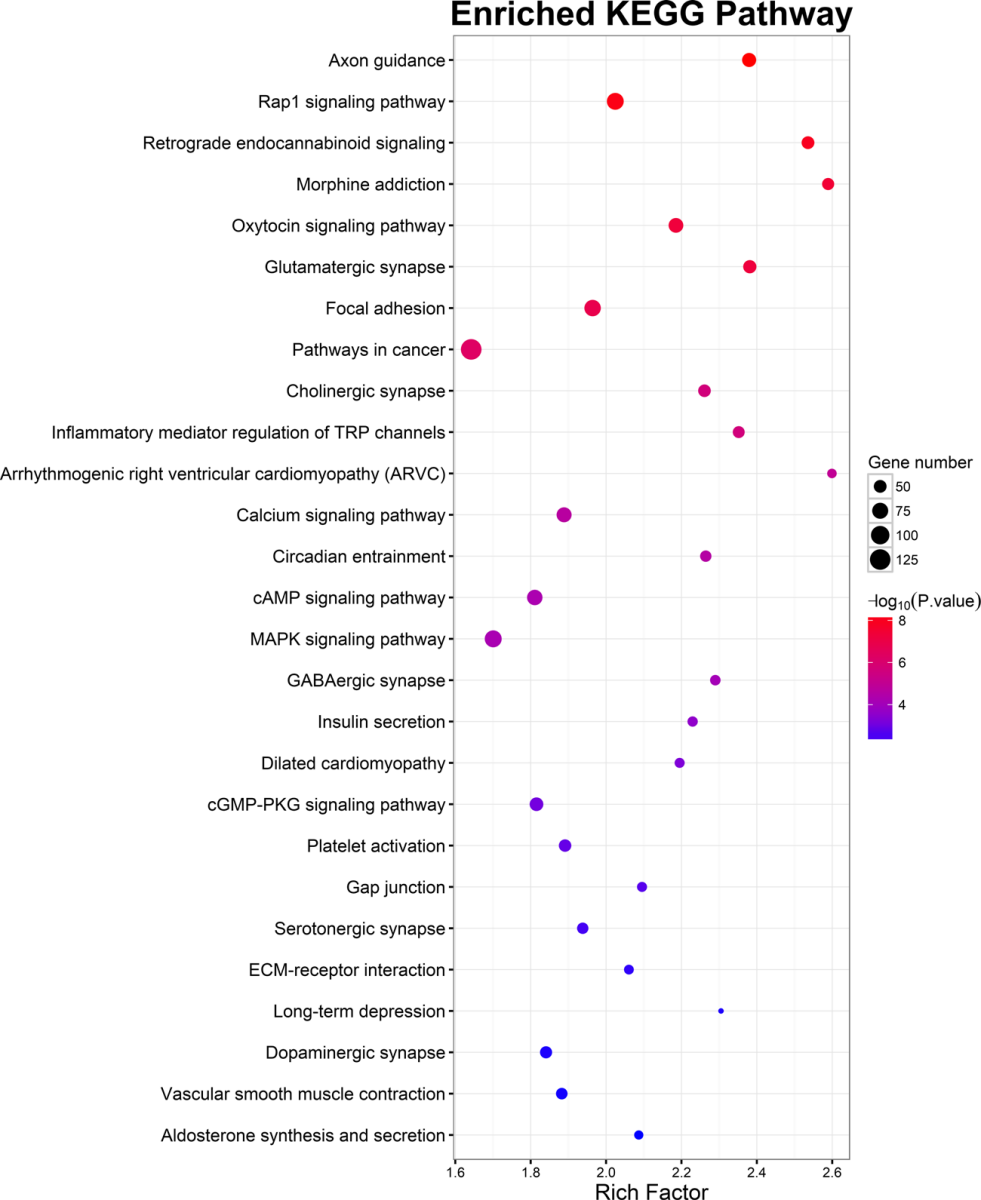
KEGG pathway enrichment analysis results for ASD PRS. Colored dots represented the corresponding KEGG pathway term, ranked according to the significance (*P* < 0.01 was selected after Bonferroni correction).

There were 14,551 SNPs and 4645 genes in ASD PRS at *P*_T__0.17, which has the smallest *P* value in the association analysis with schizophrenia. ASD PRS genes were significantly enriched in 41 GO terms (Table 3). Signal transduction, cell adhesion, and axon guidance were at the top for BPs of enriched GO terms. The CC of ASD PRS genes significantly concentrated in cell junction and neuronal structures, such as postsynaptic membrane, postsynaptic density, synapse, neuron projection, axon, and so on. ATP binding, calcium ion binding, calmodulin binding, GTPase activator activity, and actin binding were the top five enriched GO terms in MFs. ASD PRS genes were enriched in 27 KEGG pathways (Fig. 4), of which the top six pathways mainly involved in neural development (axon guidance), signal transduction (Rap1 signaling pathway), nervous system (retrograde endocannabinoid signaling and glutamatergic synapse), and neuroendocrine system (oxytocin −signaling pathway).

**Table 3:** GO terms for PRS via DAVID gene functional classification tool.

Description	GO term	Gene count	*P*	Fold enrichment	Adjusted *P*^a^
Biological process	GO:0035556~intracellular signal transduction	139	3.66E−12	1.75	2.38E−08
	GO:0043547~positive regulation of GTPase activity	179	1.12E−11	1.61	7.32E−08
	GO:0007165~signal transduction	316	1.85E−10	1.38	1.20E−06
	GO:0007155~cell adhesion	146	7.70E−10	1.61	5.02E−06
	GO:0007605~sensory perception of sound	57	2.89E−09	2.17	1.88E−05
	GO:0007169~transmembrane receptor protein tyrosine kinase signaling pathway	45	6.31E−09	2.37	4.11E−05
	GO:0007411~axon guidance	61	1.02E−07	1.94	6.67E−04
	GO:0051056~regulation of small GTPase−mediated signal transduction	53	2.55E−07	2.00	1.66E−03
	GO:0034220~ion transmembrane transport	73	4.88E−07	1.76	3.18E−03
	GO:0070588~calcium ion transmembrane transport	47	1.40E−06	2.00	9.05E−03
Cellular component	GO:0030054~cell junction	165	3.38E−17	1.86	3.29E−14
	GO:0005886~plasma membrane	958	9.87E−13	1.20	9.60E−10
	GO:0045211~postsynaptic membrane	85	3.98E−12	2.08	3.88E−09
	GO:0014069~postsynaptic density	73	3.37E−10	2.05	3.28E−07
	GO:0042383~sarcolemma	41	6.38E−09	2.49	6.21E−06
	GO:0005938~cell cortex	52	1.28E−08	2.19	1.25E−05
	GO:0005737~cytoplasm	1145	1.87E−08	1.13	1.82E−05
	GO:0005829~cytosol	752	7.92E−08	1.17	7.71E−05
	GO:0005578~proteinaceous extracellular matrix	89	9.45E−08	1.72	9.19E−05
	GO:0045202~synapse	65	2.78E−07	1.86	2.70E−04
	GO:0043235~receptor complex	50	3.53E−07	2.04	3.43E−04
	GO:0043005~neuron projection	79	4.46E−07	1.72	4.34E−04
	GO:0005911~cell–cell junction	61	1.08E−06	1.83	1.06E−03
	GO:0005887~integral component of plasma membrane	343	1.79E−06	1.25	1.74E−03
	GO:0030424~axon	73	2.23E−06	1.70	2.17E−03
	GO:0043025~neuronal cell body	96	2.30E−06	1.58	2.24E−03
	GO:0005891~voltage−gated calcium channel complex	18	3.37E−06	3.21	3.28E−03
	GO:0043197~dendritic spine	40	3.93E−06	2.07	3.82E−03
	GO:0030018~Z disc	45	3.97E−06	1.97	3.86E−03
	GO:0016324~apical plasma membrane	89	4.74E−06	1.58	4.60E−03
	GO:0005856~cytoskeleton	108	5.37E−06	1.50	5.21E−03
	GO:0030425~dendrite	99	6.85E−06	1.53	6.65E−03
	GO:0005768~endosome	72	7.71E−06	1.65	7.47E−03
Molecular function	GO:0005524~ATP binding	399	8.71E−13	1.37	1.80E−09
	GO:0005509~calcium ion binding	205	1.72E−09	1.47	3.56E−06
	GO:0005516~calmodulin binding	73	1.73E−09	1.99	3.57E−06
	GO:0005096~GTPase activator activity	92	1.05E−07	1.70	2.17E−04
	GO:0003779~actin binding	91	1.80E−07	1.68	3.72E−04
	GO:0005085~guanyl-nucleotide exchange factor activity	48	2.38E−07	2.09	4.93E−04
	GO:0005089~rho guanyl-nucleotide exchange factor activity	34	2.14E−06	2.27	4.42E−03
	GO:0044325~ion channel binding	44	3.20E−06	2.00	6.59E−03

*GO* Gene ontology.

^a^Bonferroni procedure was applied for an adjusted *P*.

## Discussion

In the present study, we found that ALFF in left amygdala was positively associated with ASD PRS, especially ASD-specific PRS. To the best of our knowledge, this is the first work that shows the correlation between spontaneous brain activity in left amygdala and common genetic risks for ASD in schizophrenia. A large body of literature shows abnormal amygdala activity in patients with schizophrenia^61,62^. The amygdala is also one of the brain regions, in which there are consistent reports of abnormality in individuals with autism^63,64^. Though aberrant activity of amygdala is a common intermediate phenotype for schizophrenia and ASD, it is unclear if this similarity is caused by same genetic factors or not. We adopted the strategy of PRS to assess the role of ASD-associated polygenic risk in schizophrenia. We calculated high-resolution ASD PRS and selected optimal ten ASD PRS that contributed to schizophrenia mostly to test the association with brain function. This may provide a promising way to understand the relationship between phenotypes and genotypes shared in mental disorders.

Interestingly, we also found that ASD PRS was negatively correlated with negative emotions in facial emotion recognition test, which further supported the correlation of PRS with ALFF in left amygdala. Obviously, amygdala plays a central role in emotion processing^65^, one of domains in social cognition^62^. Case studies of brain damaged individuals, as well as fMRI studies suggest that the amygdala is critical for accurate recognition of facial expressions^66–68^. Schizophrenia patients have deficits in facial emotion recognition, concluded from comprehensive reviews covering hundreds of studies^69,70^, particularly when processing a subset of negative emotions^24^^,^^71–73^. In the present study, ASD PRS was correlated with left amygdala activity and negative emotion, separately, indicating that common polygenic risks for ASD may lead to similar clinical symptoms (emotion recognition) through common neutral substrate (amygdala) in schizophrenia.

According to what we know, as well as several reviews^61,62,74^ in this field, most of the researches revealed patients with schizophrenia tended to show less or no activation in amygdala in response to negative emotions, such as sadness^75,76^, fear^77–79^, and anger^80^. However, in our study, we found a positive correlation between PRS and ALFF in left amygdala. One explanation for this difference is that ALFF reflects the spontaneous neural function of the brain in resting state, while previous reports evaluated the changes in the amygdala when individuals performed specific tasks. Increased spontaneous neural activity of left amygdala may lead to the deficiency of activation during the processing of a task. Mingoia and colleagues proposed an interpretation similar to ours, that an increased baseline activity might explain the diminished effect during activation due to smaller difference between baseline and activation state^81^. Moreover, several fMRI studies have reported elevated ALFF in left amygdala in schizophrenia patients^47,49,50^. In a work by Turner et al., the schizophrenia group exhibited greater ALFF in left amygdala than the HC group^47^. The same alteration of ALFF values in left amygdala in patients with schizophrenia was also found by recent reports^49,50^. A PET study also detected elevated baseline activity in amygdala in schizophrenia^82^. These findings together are in accordance with our result of a positive correlation between ASD PRS and ALFF in left amygdala.

Axon guidance was the most significantly pathway in KEGG pathway analysis of PRS genes. As the largest family of the axon guidance cues, multiple semaphorin genes, including classes 3–6 (SEMA3A/C/D/E, SEMA4B/D, SEMA5A/B, and SEMA6B/D) were found in PRS genes. Other axon guidance factors, such as netrins and slits, and their receptors were also involved in our study. Axon guidance is an essential process for proper formation of neuronal connections and network during brain development. In a global proteome analysis of healthy human amygdala, >60% of 1814 identified unique proteins were not described before in other limbic system structures, such as thalamus, olfactory bulb, or pituitary gland. Overrepresentation of human amygdaloid protein in axon guidance was observed by Reactome pathway analysis^83^, indicating the importance of axon guidance in amygdala. An integrated analysis of diverse schizophrenia associated whole-genome data sets identified several cohesive gene networks, including axon guidance, neuronal cell mobility, and synaptic function^84^.

Rap1 signaling pathway was the second most significant pathway in KEGG analysis of PRS genes. Rap1 is a small GTPase that controls diverse processes, such as cell adhesion, cell–cell junction formation, and cell polarity. In neuronal cells, Rap/Ras signaling regulates the capacity of synaptic plasticity^85^. Rap1 signaling enables plasticity and fear learning by regulating L-type calcium channels at cortico-amygdala synapses^86^. In our study, there were 83 genes enriched in this pathway, including glutamate ionotropic receptor NMDA type subunit 2A (GRIN2A) and 2B (GRIN2B).

It is interesting that oxytocin pathway genes were also enriched in our study. Neuropeptide oxytocin has been linked to social perception, cognition, and emotion behavior^87,88^. The binding of oxytocin with its specific receptor oxytocin receptor (OTR) activates a set of signaling cascades, including Gq/PLC/Ins3 pathway, the MAPK, and the RhoA/Rho kinase pathways^89^. A considerable number of studies has shown oxytocin pathway gene polymorphisms, especially OTR gene variations, are associated with neuropsychiatric disorders like autism and schizophrenia though the results have also been inconsistent^90–92^. Moreover, OTR polymorphisms also contribute to amygdala activation in schizophrenia^93^. Combined with previous studies, our results further confirm that oxytocin pathway genetic variations mediate amygdala dysfunction, and ultimately result in emotion processing abnormality in schizophrenia.

Our results should be interpreted in the context of several limitations. First, PRS is calculated based on SNPs and does not take into account all genetics risk like CNVs or rare mutations. Second, our target sample was of small size to investigate the effect of polygenic risk on brain function. Nevertheless, the discovery sample we used was the most recent and well powered. Replication of these results in additional dataset would be needed.

In a conclusion, we found that ASD PRS was correlated with ALFF in left amygdala and negative emotions in schizophrenia. Genes involved in the function and development of neural system were mainly enriched in PRS genes. Our findings indicate that polygenic risk of ASD contributes to schizophrenia by the intermediate phenotypes of left amygdala function, and thus affects emotional processes. This was a preliminary exploration to understand the relationship between phenotypes and genotypes shared in mental disorders.

## Supplementary information

Supplementary file
