# Supplementary material for: Polygenic risk for autism spectrum disorder affects left amygdala activity and negative emotion in schizophrenia
Source: Transl Psychiatry. 2020 Sep 21;10:322. doi: 10.1038/s41398-020-01001-2 (PMC7506524; doi:10.1038/s41398-020-01001-2)
Supplement: Supplementary file 1 — Supplementary file [file 41398_2020_1001_MOESM1_ESM.docx]

**Supplementary file**

Table S1. Brain regions with original p values less than 0.05 in multiple linear regression analyses between ASD PRS and ALFF of 90 AAL regions

| Polygenic risk scores | OLF.L | OLF.R | DCG.L | PCG.R | PHG.L | AMYG.L | AMYG.R | MOG.R | CAU.L | CAU.R | PUT.L | THA.L | TPOsup.L |
| --- | --- | --- | --- | --- | --- | --- | --- | --- | --- | --- | --- | --- | --- |
| *P*_T__0.09 | 0.013 | 0.015 | 0.048 | 0.037 | 0.055 | 2.66E-04 | 0.006 | 0.044 | 0.017 | 0.017 | 0.096 | 0.028 | 0.056 |
| *P*_T__0.13 | 0.020 | 0.025 | 0.031 | 0.086 | 0.039 | 2.48E-04 | 0.010 | 0.013 | 0.029 | 0.035 | 0.040 | 0.029 | 0.023 |
| *P*_T__0.14 | 0.029 | 0.040 | 0.032 | 0.118 | 0.045 | 4.55E-04 | 0.015 | 0.020 | 0.040 | 0.045 | 0.054 | 0.033 | 0.030 |
| *P*_T__0.145 | 0.038 | 0.045 | 0.040 | 0.113 | 0.036 | 3.65E-04 | 0.013 | 0.027 | 0.043 | 0.045 | 0.043 | 0.026 | 0.033 |
| *P*_T__0.15 | 0.043 | 0.045 | 0.037 | 0.111 | 0.040 | 4.20E-04 | 0.014 | 0.030 | 0.045 | 0.046 | 0.051 | 0.025 | 0.024 |
| *P*_T__0.16 | 0.047 | 0.042 | 0.043 | 0.129 | 0.053 | 7.45E-04 | 0.024 | 0.026 | 0.039 | 0.045 | 0.053 | 0.030 | 0.028 |
| *P*_T__0.165 | 0.049 | 0.040 | 0.041 | 0.159 | 0.041 | 5.97E-04 | 0.017 | 0.031 | 0.037 | 0.039 | 0.042 | 0.026 | 0.031 |
| *P*_T__0.17 | 0.049 | 0.032 | 0.043 | 0.169 | 0.042 | 6.13E-04 | 0.015 | 0.033 | 0.037 | 0.034 | 0.034 | 0.021 | 0.033 |
| *P*_T__0.175 | 0.050 | 0.031 | 0.039 | 0.152 | 0.041 | 8.11E-04 | 0.018 | 0.031 | 0.041 | 0.038 | 0.040 | 0.026 | 0.032 |
| *P*_T__0.2 | 0.037 | 0.024 | 0.078 | 0.246 | 0.065 | 1.42E-03 | 0.027 | 0.026 | 0.033 | 0.027 | 0.040 | 0.022 | 0.076 |

Brain regions listed here with original p values (*P* < 0.05) are in correspondence with data showed in Figure 2.

L, left; R, right; OLF, olfactory cortex; DCG, median cingulate and paracingulate gyri; PCG, post cingulate gyrus; PHG, parahippocampal gyrus; AMYG, amygdala; MOG, middle occipital gyrus; CAU, caudate nucleus; PUT, putamen; THA, thalamus; TPOsup, temporal pole of superior temporal gyrus.
